# Maternal Blood as a Window to the Fetal Heart: Novel Biomarkers for Early Detection of Septal Defects

**DOI:** 10.3390/biomedicines14030586

**Published:** 2026-03-05

**Authors:** Alexandru Carauleanu, Catalin M. Buzduga, Razvan I. Tudosa, Claudia Florida Costea, Anca Petruta Morosan, Alexandru Nemtoi, Emilia Patrascanu, Gina Madalina Toma, Camelia Tamas, Anca Haisan, Roxana Covali, Andrei I. Cucu, Amelian M. Bobu

**Affiliations:** 1 Faculty of Medicine, Grigore T. Popa University of Medicine and Pharmacy Iasi, 700115 Iasi, Romania; ale.carauleanu@umfiasi.ro (A.C.); claudia.costea@umfiasi.ro (C.F.C.); petruta-anca.morosan@umfiasi.ro (A.P.M.); patrascanu.emilia@umfiasi.ro (E.P.); camelia.tamas@umfiasi.ro (C.T.); anca.haisan@umfiasi.ro (A.H.); ana.covali@umfiasi.ro (R.C.); amelian.bobu@gmail.com (A.M.B.); 2 “St Maria” Emergency Children Hospital, 700309 Iasi, Romania; razvan-ionut.tudosa@email.umfiasi.ro; 3 Faculty of Medicine and Biological Sciences, Stefan cel Mare University of Suceava, 720229 Suceava, Romania; alexandru.nemtoi@usm.ro (A.N.); toma.gina24@gmail.com (G.M.T.); andrei.cucu@usm.ro (A.I.C.)

**Keywords:** congenital heart disease, prenatal diagnosis, maternal biomarkers, miRNA, lcRNA, congenital septal defects

## Abstract

Congenital heart defects (CHDs) represent the most common category of congenital malformations and constitute a significant cause of infant morbidity and mortality. Despite advances in prenatal imaging, such as fetal echocardiography, early detection remains challenging, particularly in pregnancies without identified risk factors. Recent studies suggest that maternal circulating non-coding RNAs, including microRNAs and long non-coding RNAs (lncRNAs), may serve as promising non-invasive biomarkers for the prenatal diagnosis of CHDs. Following a review of the most relevant clinical and preclinical studies, it was found that maternal circulating RNA, particularly microRNAs and lncRNAs, shows potential as non-invasive biomarkers for detecting fetal congenital heart defects. Among microRNAs, *miR-146a-5p* demonstrated the highest diagnostic accuracy for ventricular septal defects (VSDs), while panels of lncRNAs, such as *LINC00598*, *LINC01551*, and *GATA3-AS1*, exhibited high performance for atrial septal defects (ASDs). In addition, *miR-19b*, *miR-29c*, and *miR-375* were associated with both VSDs and ASDs, suggesting a shared role in septal development. However, the studies displayed variability in biomarker selection and analytical methodologies. The findings indicate that maternal circulating microRNAs and lncRNAs hold significant potential as non-invasive biomarkers for the early detection of CHDs. Nonetheless, methodological heterogeneity and small sample sizes highlight the need for standardized protocols and larger multicenter studies prior to clinical implementation. These observations support the future integration of RNA biomarkers with fetal echocardiography to enhance early CHD screening and to inform prenatal counseling.

## 1. Introduction

Congenital heart diseases (CHDs) represent the most common category of congenital defects, with a traditionally reported global incidence of approximately 8 cases per 1000 live births [1], although recent data suggest an increase to around 9–9.5 cases per 1000 births [2]. In 2021, the global prevalence of congenital heart disease in children under five years of age was estimated at over 4.18 million cases, marking an increase compared to previous years [3]. At the European level, the incidence of congenital heart disease is estimated at approximately 5.7–6 cases per 1000 births, with considerable variation across different national registries and geographic regions. For example, Norway and England/Wales have reported a decreasing trend in CHD prevalence, whereas other registries, including those from Italy and Croatia, have documented an increase in the number of reported cases [4].

Similarly, data from the United States indicate that congenital heart diseases affect approximately 1% of annual births [5]. The highest birth prevalence of congenital heart disease has been reported in Asia, with rates approaching 20 cases per 1000 live births in China [6], whereas in Africa it is estimated that approximately 500,000 newborns are affected each year, the majority of whom are from sub-Saharan Africa [7].

CHDs encompass a wide spectrum of structural defects, with the majority of cases represented by septal defects, including ventricular septal defects (≈35.6%) and atrial septal defects (≈15.4%), together accounting for over half of all cases. These are followed by outflow tract defects and obstructions, such as pulmonary stenosis (≈6.2%), coarctation of the aorta (≈3.6%), aortic stenosis (≈2.3%), aortic regurgitation (≈2.3%), and hypoplastic left heart syndrome (≈2.6%), which together constitute approximately one-quarter of cases [2]. Conotruncal defects, including tetralogy of Fallot and transposition of the great arteries, account for around 10% of cases [2,8,9], while other rarer or more complex malformations, such as patent ductus arteriosus (≈10.2%), congenital heart block (≈3.2%), or dextrocardia (≈1.0%), make up the remaining cases [2].

With a complex and incompletely understood etiology [8], congenital heart malformations remain a major cause of morbidity and mortality [9], highlighting the need for early screening and detection, even during the intrauterine period.

Given that the most common congenital heart defects are represented by septal defects, this review aims to synthesize the available literature on circulating non-coding RNA biomarkers in maternal blood, particularly microRNAs and long non-coding RNAs, involved in the prenatal diagnosis of these conditions and to examine the latest techniques for detecting and quantifying maternal RNAs and their potential clinical applicability as a complementary tool to fetal echocardiography.

## 2. Heart Evolution

**Figure 1:**
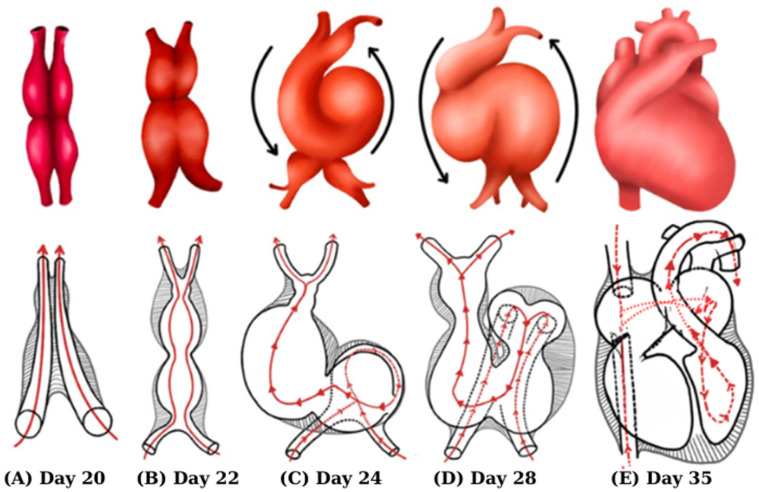
Heart evolution (figure created by R. Tudosa). (**A**) Endothelial tubes begin to fuse to form primary heart tube, which is divided into incipient chambers by sulci. (**B**) Myocardium invests endocardial heart tube and forms cardiac jelly. Heart begins to beat. (**C**) Heart continues to elongate and starts to bend. (**D**) Bending continues as ventricle moves caudally and atrium moves cranially. (**E**) After heart looping is complete, septation, cardiac remodeling, and valve formation begin, ensuring chamber alignment and separation of systemic and pulmonary circulations; disruption of these processes can lead to complex congenital heart malformations. Red arrow: systemic circulation. Red dashed arrow: blood in systemic circulation after the cardiac chambers begin to form.

Cardiogenesis underlies the development of congenital heart diseases. Human heart morphogenesis begins early post-conception, with major structures forming between days 15–45 and most achieving their final configuration by day 30 [8,10]. Heart development progresses through four overlapping stages [11,12]: early cardiogenesis (the formation of the first heart field and primitive heart tube) [13,14,15], morphogenesis (chamber and outflow tract formation via the first and second heart fields) [13,14,15,16,17,18], septation and remodeling (valve and septa formation, chamber alignment, and circulation separation) [13,16,19,20], and fetal maturation (myocardium, valves, conduction system, and coronary circulation) [16,21,22,23,24]. Disruption at any stage can lead to congenital heart malformations. Figure 1 summarizes the key stages of heart development, from the formation of the primary heart tube to chamber septation and the establishment of systemic and pulmonary circulation.

## 3. Atrial and Ventricular Septation

Atrial and ventricular septation represent critical steps for the separation of circulations and the alignment of the chambers, being achieved through the interaction of the endocardial cushions, the muscular and membranous septa, and the outflow tract structures [11,13,16,25]. Atrial septation begins at Carnegie Stage 12 (CS12) with the formation of the primary septum and continues through the growth and remodeling of the endocardial cushions and the dorsal mesenchymal protrusion [16,26]. Ventricular septation initially involves primitive muscular portions, followed by the development of the membranous septum through the fusion of the atrioventricular cushions with the outflow tract ridges, completing the separation of the ventricular circulations by CS18 [16,27,28].

The precise regulation of gene expression is essential for the coordinated formation of endocardial cushions and muscular and membranous septa and the alignment of the outflow tracts. Recent evidence highlights the critical role of noncoding RNAs (ncRNAs) and transcription factors, such as *GATA4*, in orchestrating these processes [29]. Alterations in *GATA4* expression or hypermethylation of the gene have been associated with congenital heart defects, including ventricular septal defects (VSDs) and atrial malformations [30,31,32].

Non-coding RNAs (NcRNAs), including microRNAs (miRNAs) and long noncoding RNAs (lncRNAs), are key regulators of cardiac development, controlling cardiomyocyte proliferation, differentiation, and septation [29]. *MiR-335-3p/5p* stimulates the expression of mesodermal and cardiac genes [33]. *MiR-1* promotes the exit of cardiac progenitor cells from the cell cycle and their differentiation into cardiomyocytes, and it is also involved in the development of myocardial sarcomeres and the conduction system. [34,35]. *MiR-133* inhibits cardiac differentiation, while miR-133a-1/2 coordinate cardiomyocyte proliferation and differentiation in the posterior segment of the cardiac tube, and their dysregulation can lead to VSDs [29,36]. Other miRNAs, including *miR-17-92*, *miR-322/-503*, *miR-218*, *miR-27b*, *miR-499*, and *miR-302-367*, regulate cardiomyocyte migration, differentiation, and proliferation, and their dysregulation is associated with thinning of the ventricular wall and septation defects [29,37,38,39].

LncRNAs modulate the specification and differentiation of cardiac progenitors through interactions with transcription factors and epigenetic complexes. *Linc1405* activates *Mesp1* to drive mesodermal specification [40], *Braveheart (Bvht)* and *CARMEN* promote cardiac development via *PRC2* [29,41], and *CARMA* regulates cardiomyocyte differentiation by modulating the Notch pathway [42]. Other lncRNAs, including *Platr4*, *Novlnc6*, *Moshe*, and *Uph*, control the expression of genes critical for proliferation and differentiation, and their dysregulation is associated with VSDs [29,43,44]. Additionally, *BANCR* regulates cardiomyocyte migration, and its loss can lead to increased heart size [45].

These findings suggest that ncRNAs are not only regulators of normal atrial and ventricular septation but also potential biomarkers for congenital heart disease. Incorporating ncRNA profiles into early developmental studies may provide insights into the molecular mechanisms of septum formation and identify novel targets for diagnostic and therapeutic strategies in patients with septal defects.

## 4. Cardiac Septal Defects: Classification

In the context of these finely regulated cardiac septation processes, any disruption of the embryonic mechanisms described above, whether caused by genetic, epigenetic, or environmental factors, can interfere with normal septal development, leading to the occurrence of septal defects [46,47]. Consequently, the persistence of openings between the right and left atria after birth clinically manifests as atrial septal defects (ASDs) and ventricular septal defects (VSDs), whose location and severity depend on the septal segment affected during embryogenesis.

### 4.1. Atrial Septal Defects (ASDs)

**Figure 2:**
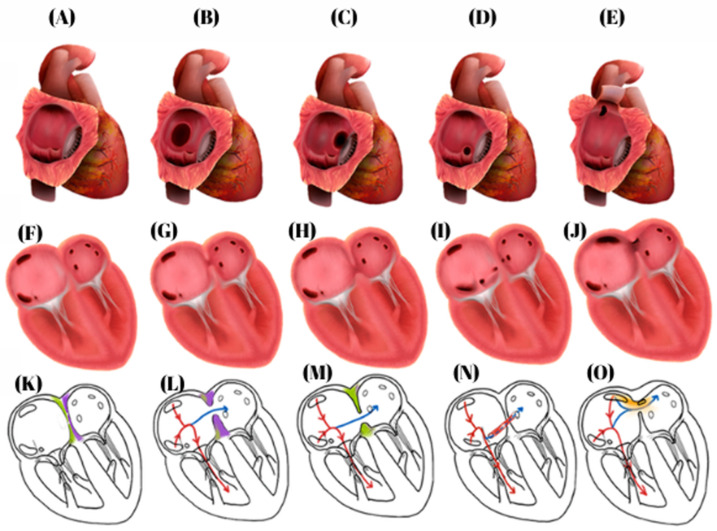
Atrial septal defects (figure created by R. Tudosa). (**A**) Normal heart, visualized in cross-section at the level of the right atrium. (**B**) Ostium secundum, visualized in cross-section at the level of the right atrium. (**C**) Ostium primum, visualized in cross-section at the level of the right atrium. (**D**) Coronary sinus septal defect, visualized in cross-section at the level of the right atrium. (**E**) Sinus Venosus type defect is located at the junction between the right atrium and the superior vena cava, visualized in cross-section at the level of the right atrium. (**F**) Normal heart, visualized in four-chamber view. (**G**) Ostium secundum, visualized in four-chamber view. (**H**) Ostium primum, visualized in four-chamber view. (**I**) Coronary sinus septal defect, visualized in four-chamber view. (**J**) Sinus venosus type defect is located at the junction between the right atrium and the superior vena cava, visualized in four-chamber view. (**K**) Normal heart, schematic view. (**L**) Ostium secundum, schematic of the atrial defect and blood flow. (**M**) Ostium primum, schematic of the atrial defect and blood flow. (**N**) Coronary sinus septal defect, schematic of the atrial defect and blood flow. (**O**) Sinus Venosus type defect is located at the junction between the right atrium and the superior vena cava, schematic of the atrial defect and blood flow. Red arrow: systemic circulation; Blue arrow: the direction of blood flow through the atrial septal defect.

Five types of atrial septal defects are recognized and illustrated in Figure 2: (1) ostium secundum ASDs, the most common form, accounting for 80–90% of cases [26,48,49]; (2) ostium primum ASDs, part of the atrioventricular septal defect spectrum [26,48,50]; (3) superior sinus venosus ASDs, included within the 2–10% of sinus venosus defects [48,49,51]; (4) inferior sinus venosus ASDs, a rare variant of this group [48,52]; and (5) coronary sinus defects, the least common subtype [49,53].

### 4.2. Ventricular Septal Defects 

**Figure 3:**
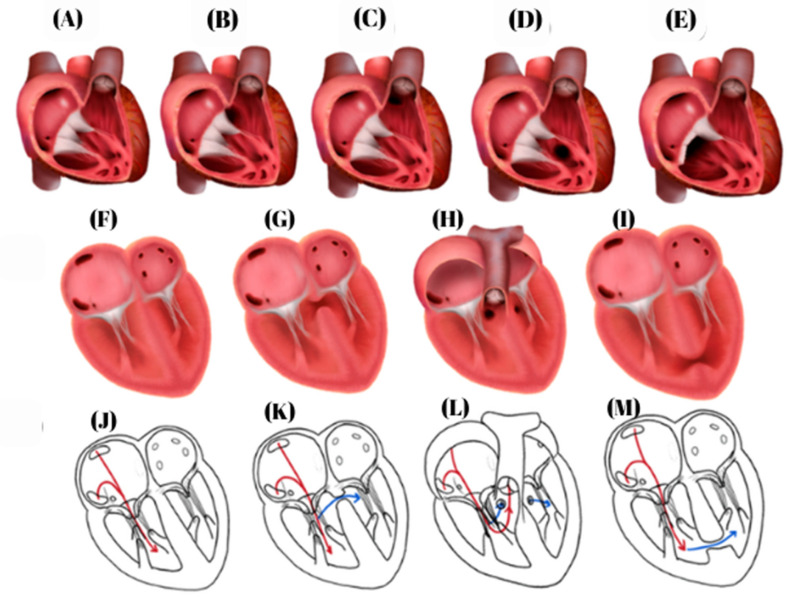
Types of ventricular septal defects (figure created by R. Tudosa). (**A**) Normal Heart. (**B**) Perimembranous ventricular septal defect. (**C**) Infundibular ventricular septal defect. (**D**) Muscular ventricular septal defect. (**E**) Inlet (atrioventricular canal) septal defect. (**F**) Normal heart, four-chamber view. (**G**) Perimembranous ventricular septal defect, four-chamber view. (**H**) Infundibular ventricular septal defect, four-chamber view. (**I**) Muscular ventricular septal defect, four-chamber view. (**J**) Normal Heart, schematic view. (**K**) Perimembranous ventricular septal defect, schematic of the ventricular defect and blood flow through the ventricular septal defect. (**L**) Infundibular ventricular septal defect, schematic of the ventricular defect and blood flow through the ventricular septal defect. (**M**) Muscular ventricular septal defect, schematic of the ventricular defect and blood flow through the ventricular septal defect. Red arrow: systemic circulation; Blue arrow: the direction of blood flow through the septal defect.

VSDs are classified into four main anatomical types [54], illustrated in Figure 3: (1) perimembranous VSDs, the most common form, accounting for approximately 80% of cases [46,55]; (2) inlet (atrioventricular canal) VSDs, representing about 8% of cases [56,57,58]; (3) infundibular (outflow tract) VSDs, a less common subtype accounting for approximately 6% of cases, with higher prevalence in Asian populations [59,60]; and (4) muscular VSDs, located within the trabecular septum and variable in number and distribution [27,61,62,63].

In addition to these classic forms, the Gerbode defect represents a rare (<1%) type of interventricular communication between the left ventricle and right atrium [64,65,66].

## 5. Prenatal Screening

The early detection of severe congenital heart malformations during pregnancy represents a crucial tool for the psychological counseling of an expectant mother and for informed therapeutic decision-making, providing families with adequate time to evaluate prognosis, perform prenatal genetic testing, and, in severe cases, make decisions regarding pregnancy management [67,68,69].

Currently, the primary instrument for the prenatal screening of congenital heart defects is fetal ultrasound, which allows a detailed assessment of the fetal heart and great arteries [70]. The importance of this screening is further underscored by the fact that nearly half of pregnancies are unplanned, and risk factors for congenital heart disease are identified in only 10% of cases [71]. Even in low-risk populations, fetal echocardiography can detect up to 40% of cardiac malformations when abnormalities are suspected based on routine obstetric ultrasound [72].

### 5.1. The Role of Maternal Biomarkers in the Prenatal Diagnosis of CHD 

In this context, comprehensive prenatal diagnosis and studies on embryogenesis are becoming increasingly important. Currently, there are no biomarkers used in clinical practice for the detection of CHDs during pregnancy [73]. Current clinical strategies rely primarily on fetal echocardiography; however, this is available only in specialized centers, and pregnancies without identified risk factors are not systematically evaluated. Moreover, diagnostic accuracy largely depends on the operator’s experience and training [73,74].

For this reason, the detection of biomarkers in maternal blood represents a highly promising and potentially transformative approach in the prenatal diagnosis of CHD [73]. When combined with fetal echocardiography, this strategy may have the potential to improve screening accuracy and facilitate earlier referral to specialized evaluations. However, it is important to emphasize that, at present, no maternal biomarker has been validated for routine clinical use in prenatal CHD diagnosis. The available evidence remains exploratory, is largely based on small cohorts, and requires further validation in larger, well-designed studies.

This complementary approach may provide additional insights into the pathophysiological mechanisms underlying CHDs and could, in the future, support risk stratification and the planning of early targeted interventions. Nevertheless, its clinical implementation remains investigational, and its broader application will depend on robust prospective validation [75].

### 5.2. Biomarkers

In this context, research interest has focused on the identification of non-invasive molecular biomarkers capable of reflecting the complex processes involved in cardiac embryogenesis. Human genetics studies have demonstrated that both hereditary and sporadic forms of congenital heart disease are associated with alterations in genes encoding transcription factors essential for the morphogenesis of the ventricular septum and outflow tract during heart development [76,77].

However, the phenotypic expression of these anomalies is not determined solely by genetics but is profoundly influenced by epigenetic mechanisms, such as DNA methylation and non-coding RNAs (ncRNAs) [77,78]. Among these, microRNAs (miRNAs) represent a highly conserved class of non-coding RNAs involved in the post-transcriptional regulation of gene expression, with key roles in the cell cycle, cardiomyocyte differentiation, and normal cardiac development [77,79]. By modulating transcription factors involved in ventricular septum formation, miRNAs influence heart morphogenesis and pathophysiological processes such as myocardial hypoxia and cardiac remodeling, all of which are closely linked to the onset, progression, and severity of ventricular septal defects [79,80].

A major advantage of miRNAs is their stability in the extracellular environment, as they are protected from degradation through encapsulation in lipid vesicles or association with protein and lipoprotein complexes, allowing reliable detection in maternal blood [81,82,83]. These characteristics support the potential of miRNAs as circulating biomarkers for the prenatal screening of congenital heart diseases, opening new avenues for early diagnosis and for understanding the molecular mechanisms involved in the development of cardiac malformations.

In addition to epigenetic biomarkers, recent research has also explored proteomic and protein-based biomarkers capable of reflecting functional and structural changes associated with fetal cardiac development. In this context, circulating proteins detectable in maternal blood have been investigated, including exosomal lactoferrin [84,85] and complement regulatory factors [86], as well as panels of cytoskeletal proteins involved in cellular organization and tissue remodeling [73,87]. Profiling these molecules provides complementary information on the molecular mechanisms underlying the pathogenesis of congenital heart diseases and outlines an emerging direction in prenatal screening. The synthesis of these biomarkers is presented in Table 1.

## 6. Methods

This review was conducted in accordance with the PRISMA 2020 guidelines (Supplementary File S1). A comprehensive search was performed in three electronic databases: PubMed, Scopus, and Web of Science. The search covered publications from January 2010 to October 2025. Search terms were defined based on a preliminary review and domain expertise and included microRNA, lncRNA, heart defects, congenital, congenital heart malformations, ventricular septal defect, atrial septal defect, prenatal diagnosis, and non-invasive prenatal testing.

**Figure 4:**
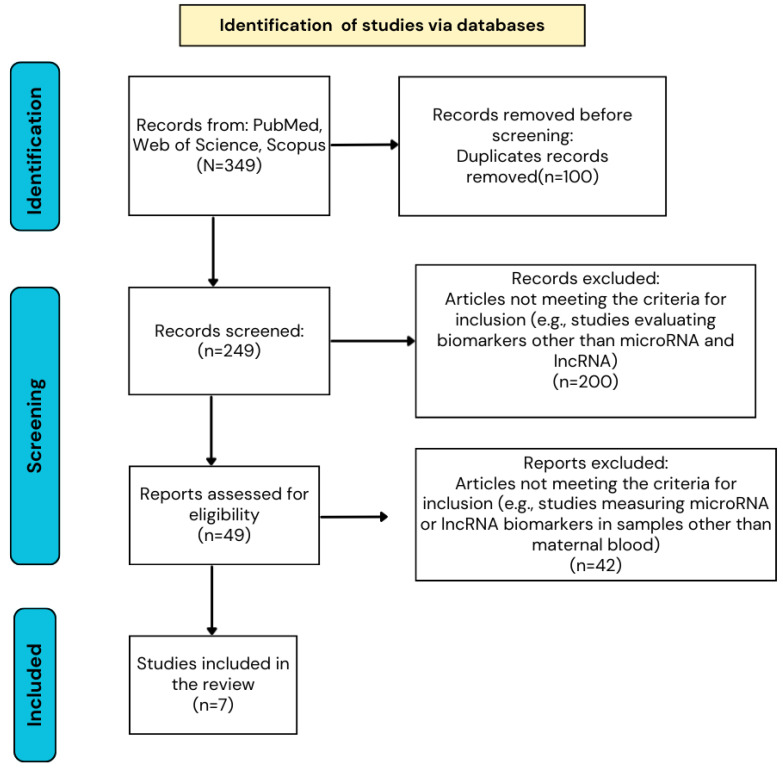
Prisma flowchart.

Only original articles published in English and available in full text were included. Reviews, case reports, case series, opinions, and publications in other languages were excluded. After removing duplicates, titles and abstracts were screened, and full texts were assessed according to predefined inclusion and exclusion criteria. Articles measuring microRNA or lncRNA biomarkers in samples other than maternal blood were excluded. Ultimately, seven studies were included in the review (Figure 4). Sample sizes ranged from 13 to 182 participants, with a balanced distribution between study and control groups in most studies. CHD diagnosis was primarily based on fetal echocardiography, occasionally complemented by postnatal surgical intervention or autopsy. The most frequently studied type of CHD was ventricular septal defect.

Due to significant heterogeneity in study design, ncRNA detection methodologies, sample types, outcome definitions, and reporting formats, a formal meta-analysis was not feasible. Therefore, a structured narrative synthesis was performed to present the results.

## 7. Results and Discussion

NcRNAs are RNA molecules that do not encode proteins but play essential roles in gene expression regulation and fundamental cellular processes. To date, approximately 17 categories of ncRNAs have been identified, including transfer RNA (tRNA), ribosomal RNA (rRNA), small nucleolar RNA (snoRNA), microRNA (miRNA), small interfering RNA (siRNA), piwi-interacting RNA (piRNA), circular RNA (circRNA), and long non-coding RNA (lncRNA), among others [93].

Among these, miRNAs and lncRNAs have attracted particular interest in cardiac studies, as they regulate cardiomyocyte proliferation, differentiation, and survival, being involved in normal heart development and the pathogenesis of congenital heart diseases [94]. Given the relative stability of non-coding RNAs in biological fluids, as well as their tissue-specific and functional characteristics, these molecules represent promising candidates for the development of non-invasive biomarkers capable of reflecting the molecular processes involved in the pathogenesis of congenital septal defects [95].

### 7.1. Preclinical Studies on Prenatal Biomarkers

#### 7.1.1. Preclinical Studies on MicroRNA

MicroRNAs actively participate in cardiac development, and their dysregulation may contribute to the onset of congenital heart diseases [76,77]. The absence of the enzyme Dicer, responsible for miRNA processing, can lead to the abnormal formation of the outflow tract and cardiac chambers in mammals, highlighting the essential role of miRNAs in heart development [77,80,96].

A preclinical study conducted on pregnant rats whose fetuses exhibited ventricular septal defects revealed the differential expression of multiple microRNAs [77]. MiR-1-3p and miR-1b were downregulated in the myocardium but upregulated in amniotic fluid and circulation, suggesting their release from cardiomyocytes and involvement in the direct regulation of solute carrier family 8 member 1/sodium–calcium exchanger 1 *(SLC8A1/NCX1)*, a calcium transporter essential for cardiomyocyte survival and ventricular septum morphogenesis [77,97,98,99]. *MiR-293-5p* showed a similar profile, while *miR-206*, *miR-184*, and *miR-15b-5p* were increased in amniotic fluid and serum, being involved in regulating cardiomyocyte proliferation, differentiation, apoptosis, and response to oxidative stress [77]. 

In another preclinical study, rats homozygous for the deletion of the *miR-1-2* locus exhibited severe ventricular septal defects, although the external anatomy of the embryonic heart appeared normal. Molecular analyses demonstrated that the absence of *miR-1-2* led to increased protein levels of the transcription factor Hand2 without altering messenger RNA (mRNA) levels, confirming the role of *miR-1-2* in regulating the translation of proteins critical for cardiomyocyte morphogenesis [100]. Additionally, *miR-92* deficiency was associated with the occurrence of ventricular septal defects (VSDs) in rat embryos, underscoring its critical role in normal heart development [101].

In another murine preclinical model of streptozotocin-induced pregnancy, maternal diabetes significantly increased the incidence of fetal cardiac developmental defects, including VSDs [102]. The profiling of circulating maternal exosomal microRNAs revealed significant alterations in the expression of multiple miRNAs involved in cardiogenesis (e.g., *miR-133*, *miR-30*, *miR-99*, *miR-23*), suggesting that disruptions in maternal exosomal signaling contribute to the development of VSDs [102,103]. Furthermore, diabetic maternal exosomes crossed the placental barrier and increased the risk of cardiac defects when injected into normal pregnancies, supporting their mechanistic role and potential as biomarkers in the preclinical context [102].

#### 7.1.2. Preclinical Studies on LncRNA

LncRNAs affect cardiac development by modulating gene expression at both pre- and post-transcriptional levels, disrupting cardiomyocyte differentiation and critical genetic signaling, thereby contributing to the occurrence of congenital heart defects [104].

An example is the long non-coding *RNA Upperhand (Uph)*, studied in mouse embryos. Blocking *Uph* transcription abolished *Hand2* expression in the right ventricle and outflow tract, resulting in right ventricular hypoplasia, single ventricle formation, and embryonic lethality. Its role does not depend on the mature transcript but on the active maintenance of the *Hand2* super-enhancer, facilitating the binding of *GATA4* (a cardiac transcription factor involved in the regulation of gene expression, including RNA transcriptional elongation) and RNA polymerase II elongation, thus acting as a cis-regulator of *Hand2* [105]. 

Thus, Uph functions as a cis-regulator of *Hand2*, being critical for the proper formation of the right ventricle. This mechanism is directly linked to the study by Misra et al., which demonstrated that the *Gata4 G295S mutation*, equivalent to the human *G296S mutation*, causes functional deficits in cardiomyocyte proliferation in vivo. Mice homozygous for *Gata4 G295S* exhibited early embryonic lethality and thin ventricular myocardium, associated with the reduced expression of *Gata4* target genes. Even heterozygous *Gata4 G295S* mice presented subtle defects, such as persistent atrial communications and semilunar valve stenoses, along with reduced cardiomyocyte proliferation in atria and ventricles during intermediate embryonic stages [106].

### 7.2. Clinical Studies on Prenatal Biomarkers

In recent years, accumulated evidence has demonstrated that epigenetic modifications are essential regulators of gene expression and play a critical role in coordinating and maintaining normal heart development [107]. The potential of circulating maternal microRNAs and lncRNAs as biomarkers for fetal CHD detection has been investigated in seven studies included in this review. Notably, six of the seven studies included in this review were conducted in China. While this geographic concentration may appear surprising, it aligns with the fact that China reports one of the highest incidences of congenital heart disease worldwide, which likely explains the focus of prenatal biomarker research in this population. The studies employed various designs, including case–control, retrospective, and prospective cohorts, with sample sizes ranging from 13 to 182 participants. Most studies relied on fetal echocardiography for CHD diagnosis, with some confirming results postnatally through surgical intervention or autopsy.

The VSD was the most frequently studied CHD type, while a few studies also included ASDs. Maternal blood samples were predominantly collected during the second trimester, and RNA was extracted from serum, plasma, or exosomes and analyzed using quantitative real-time polymerase chain reaction (qRT-PCR), microarray, NanoString, or high-throughput sequencing. Several studies reported panels of up- or downregulated microRNAs or lncRNAs with promising diagnostic performance, including area under the curve (AUC) values up to 0.997.

As reported by Jin et al., the microRNA *hsa-miR-146a-5p* exhibited the highest diagnostic value among all biomarkers analyzed in the review, with a sensitivity of 98.1% and a specificity of 98.1%, highlighting its strong potential as a biomarker for fetal VSD detection [108]. Xi et al. identified *miR-122-5p* and *miR-3195*, both downregulated, as potential biomarkers for fetal CHD detection. Individually, these microRNAs showed AUC values of 0.702 and 0.675, respectively, while their combination improved diagnostic performance to an AUC of 0.729 [109].

In the study by Zhu et al., 2013, *miR-19b*, upregulated, demonstrated the highest sensitivity and specificity among the analyzed microRNAs (74.1% and 77.8%, respectively), with an AUC of 0.7995. The use of a combined panel consisting of *miR-19b*, *miR-22*, *miR-29c*, and *miR-375* increased the AUC to 0.813, highlighting the advantage of a multiplex approach for diagnosis [110].

In the study by Wang et al., three maternal lncRNAs (*LINC00598*, *LINC01551*, and *GATA3-AS1*) were identified as potential biomarkers for fetal VSD detection. Individually, these lncRNAs exhibited AUC values of 0.852, 0.864, and 0.957, respectively, and combining the three lncRNAs significantly enhanced diagnostic performance, reaching an AUC of 0.994, suggesting the high potential of lncRNA panels for early diagnosis [104]. 

Regarding ASDs, the study conducted by Gu et al., which included the largest number of ASD cases reported at that time (*n* = 18), identified maternal lncRNAs *AA709223* and *BX478947* as potential biomarkers for fetal ASDs. Their reduced expression in maternal blood was significantly associated with the presence of atrial septal defects, supporting the diagnostic value of circulating maternal lncRNAs in prenatal ASD screening [111]. Additionally, the role of maternal microRNAs in association with atrial septal defects was evaluated in the study by Zhu et al., in which analysis of maternal serum revealed the significant overexpression of *miR-19b*, *miR-29c*, and *miR-375* in association with ASDs, supporting their potential as circulating maternal biomarkers for fetal ASDs. Notably, *miR-19b* and *miR-29c* were overexpressed in both ASDs and ventricular septal defects (VSDs), suggesting a shared role in septal defect pathogenesis, whereas *miR-19b* stood out due to its association with both ASDs and VSDs, indicating a potentially sensitive but non-specific septal biomarker [110].

The following section summarizes the main clinical studies that have investigated circulating maternal biomarkers, including miRNAs and lncRNAs, associated with septal defects (VSDs and ASDs) and other forms of CHD, with emphasis on study design and diagnostic performance (Table 2).

Although congenital heart defects (CHDs) represent the most common congenital pathology in newborns and the majority can be detected prenatally, some forms remain undiagnosed. In the absence of clinically validated maternal serum biomarkers, the development of complementary markers to fetal echocardiography could significantly enhance prenatal screening and reduce CHD-related mortality [114].

Based on the analysis of studies on prenatal biomarkers for septal defects, evidence suggests that both miRNAs and lncRNAs may reflect molecular alterations associated with fetal cardiac development and hold potential as non-invasive markers for the early detection of VSDs and ASDs. However, there is considerable variability among published studies related to sample collection and processing methods, quantification techniques, and cohort heterogeneity. Most studies were conducted in Asian populations, highlighting the need to expand research across other continents and diverse ethnic groups to validate the universal applicability of these biomarkers and to develop standardized protocols for prenatal screening.

In the reviewed studies, maternal serum was predominantly collected in the second trimester. Similar to other second-trimester biomarkers, such as elevated human chorionic gonadotropin and increased nuchal fold, these maternal serum markers are associated with CHD risk, and their combination with fetal echocardiography at the same gestational period may improve detection specificity [115,116,117].

Although circulating miRNAs and lncRNAs show promise as non-invasive biomarkers for the prenatal detection of septal defects, their clinical utility remains limited by variability across studies in populations, sample processing, and detection methods. Compared with other classes of molecular biomarkers, circulating miRNAs have a relatively prolonged half-life, estimated between approximately 4 and 24 h, which supports their stability and suitability for use as robust, non-invasive markers. Emerging evidence suggests that circulating lncRNAs exhibit comparable stability, with half-lives similar to those of miRNAs (~17–18 h), further supporting their potential as non-invasive biomarkers despite less well-characterized mechanisms of extracellular protection [118,119]. In this context, the use of circulating miRNAs as early biomarkers of cardiovascular risk has been increasingly explored. These molecules are detectable in accessible fluids, stable in circulation, and can reflect pathological changes before clinical symptoms appear [120].

Stability studies further support their use as reliable biomarkers: *miR-15b*, together with *miR-16, miR-21, miR-24,* and *miR-223*, maintains consistent serum and plasma levels even with delayed sample processing, likely due to their encapsulation in extracellular vesicles and association with Argonaute proteins or lipoproteins, making them robust and reproducible markers [120].

Preclinical models showed that *miR-15b-5p* (encoded by the *miR-15b* gene) was elevated in the serum and amniotic fluid of fetuses with ventricular septal defects, alongside *miR-206* and *miR-184*, suggesting roles in cardiomyocyte proliferation and survival [121]. Consistently, the Helsinki Birth Cohort Study demonstrated higher serum *miR-15b-5p* levels in men born to mothers with elevated BMI, independent of current adiposity, supporting a potential fetal programming effect on cardiovascular risk. Experimental models further revealed that *miR-15b-5p* overexpression impairs fatty acid oxidative metabolism and increases susceptibility to ischemia–reperfusion injury, while cardiac release, including in extracellular vesicles, points to inter-organ communication [122]. Collectively, these findings indicate that *miR-15b-5p* may serve both as a biomarker of suboptimal prenatal exposure and as a mechanistic mediator of later cardiovascular vulnerability.

Zhu et al. identified *miR-19b*, *miR-22*, *miR-29c*, and *miR-375* as promising biomarkers, reporting an area under the curve (AUC) of 0.813, indicating good diagnostic accuracy [110]. Similarly, Gu et al. reported high AUC values of 0.920 for *miR-142-5p*, *miR-1275*, *miR-4666a-3p*, and *miR-3664-3p*, which effectively discriminated cases from controls [112]. Furthermore, Wang et al. highlighted three maternal lncRNAs—*LINC00598*, *LINC01551*, and *GATA3-AS1*—as potential biomarkers for the detection of fetal VSDs [105].

Notably, *miR-375* was reported as upregulated in two independent studies, Zhu et al. 2013 [110] and Turan et al. 2019 [113], conducted on cohorts from different continents, associated with VSDs and ASDs, respectively. This replication across diverse populations suggests that *miR-375* may represent a more specific and robust biomarker for septal defects, providing a promising starting point for future multicenter validation studies and the development of universal prenatal CHD screening panels. Mechanistic studies using P19 embryonal carcinoma cells further support its role in cardiac development: the overexpression of *miR-375* inhibits cardiomyocyte proliferation and differentiation while promoting apoptosis [123]. These effects are mediated through the direct targeting of *NOTCH2 (Notch receptor 2)* and negative modulation of the Notch signaling pathway, including *DLL1 (Delta-like ligand-1)* and *HES1 (Hes Family BHLH Transcription Factor 1)*, which are essential for the formation of the septa, atrioventricular canal, and valves [123,124]. Collectively, these findings indicate that *miR-375* plays a critical role in cardiac morphogenesis, and its dysregulation may contribute to the pathogenesis of congenital septal defects, highlighting its potential both as a prenatal biomarker and a therapeutic target. In contrast, *miR-29c* was identified in Zhu et al. (2013) and included in a two-phase internally validated panel for VSDs and ASDs [110], but it has not yet been replicated in other studies, highlighting the need for external validation before its broader clinical application. Mechanistic studies in P19 embryonal carcinoma cells and zebrafish embryos suggest that *miR-29c* similarly influences cardiac development by promoting cardiomyocyte differentiation and apoptosis while inhibiting proliferation, likely via the suppression of the *Wnt4/β-catenin* signaling pathway [125]. In zebrafish, the overexpression of *miR-29c* leads to dose-dependent cardiac abnormalities, including bradycardia, pericardial edema, and looping defects, further supporting its potential involvement in septal morphogenesis [126], although additional validation is required to confirm its utility as a prenatal biomarker.

Clinical studies on circulating maternal biomarkers for the prenatal detection of septal defects (VSDs and ASDs), as summarized in Table 2, have highlighted substantial heterogeneity related to the sample type, RNA extraction method, and detection platform, directly affecting result comparability. Most studies used maternal serum (Gu et al., 2019, Zhu et al., 2013, Turan et al., 2019) [110,112,113], which provided relatively good stability for circulating microRNAs and lncRNAs and enabled high AUC values for *miR-19b*, *miR-29c*, and *miR-375*; for example, the combined panel in Zhu et al., 2013, achieved an AUC of 0.813 [110]. In Jin et al., 2021 [108], exosomes isolated from serum allowed more consistent concentrations and exceptional sensitivity and specificity for *hsa-miR-146a-5p* (AUC 0.997, sensitivity 98.1%, and specificity 98.1%), suggesting that exosome isolation can amplify the circulating miRNA signal. In contrast, studies using plasma (Gu et al., 2016) [111] reported slightly lower AUCs and sensitivity for both microRNAs and lncRNAs, likely due to dilution and protein interference, although the Arraystar + qRT-PCR platform demonstrated diagnostic relevance for lncRNAs (e.g., the combined *AA709223* and *BX478947* panel). Extraction kits and specific methods (mirVana PARIS for serum, TRIzol LS for plasma, Ribo™ Exosome Isolation for exosomes) influenced RNA yield and signal reproducibility; for example, *miR-29c* was detected only in serum using mirVana PARIS [110], whereas *miR-375* was identified in both serum and exosomes, demonstrating greater robustness [110,113]. For lncRNAs, serum processed via qPCR (Wang et al., 2023) [105] enabled a combined panel with an AUC of 0.994, compared with plasma + microarray (Gu et al., 2016) [111], highlighting that the sample type and detection method directly impact diagnostic performance. These findings underscore the need for standardized protocols for sample collection, RNA extraction, and analysis to enable the multicenter validation and universal clinical application of prenatal biomarkers for CHDs.

Most studies included small and heterogeneous cohorts, limiting the statistical power and generalizability of the findings. Additionally, the populations studied were often restricted to specific regions or ethnic groups, which may reduce applicability to broader populations.

However, the interpretation of these findings must be approached with caution, given the variability among available studies. This includes differences in the types of miRNAs and lncRNAs investigated, the populations studied, sample sizes, sample collection methods, and detection and quantification techniques. MicroRNA expression can be influenced by both genetic and environmental factors, and identifying consistent miRNA and lncRNA profiles across diverse cases of CHDs remains a significant challenge.

The clinical significance of circulating miRNAs and lncRNAs for CHD detection remains an active area of research. Although these biomarkers are promising, several challenges and limitations must be addressed before their integration into clinical practice. CHDs encompass a broad spectrum of structural cardiac malformations, each with distinct genetic and molecular characteristics, complicating the identification of universal markers.

## 8. Conclusions

The results of this review suggest that certain maternal microRNAs and lncRNAs could serve as non-invasive biomarkers for the early detection of fetal CHDs. However, the studies identified different biomarkers and employed varied methodologies, which, combined with small sample sizes and the heterogeneity of CHDs, highlight the need for caution in interpretation. Currently, these findings are not ready for clinical application, and larger, standardized studies focusing on specific CHD subtypes are required to validate them and explore their clinical utility. Until such validation is achieved, proteomic biomarkers, including those related to the cytoskeleton, represent viable alternatives and, together with routine fetal echocardiography, can significantly improve the early detection of congenital heart defects, offering a combined approach that may enhance diagnostic accuracy and guide prenatal management.

## Figures and Tables

**Table 1:** Maternal serum biomarkers associated with the detection of fetal septal defects.

Category of Biomarkers	Subcategory/Biological System	Specific Biomarker	Study
Proteomics	Cytoskeletal Proteins	*LMNA*, *FLNA*, *TPM4*, *ACTG1*	Chen et al., 2016 [73]
	Fetal Growth and Remodeling Protein	*POSTN*, *PAPPA*	Xi et al., 2023 [88]
	Exosome-Associated Proteins	*Lactoferrin*	Li et al., 2022 [85]
	Complement System	*CFHR4*, *MBL2*	He et al., 2022 [86]
Metabolites	Amino Acids	Glycine	Qiao et al., 2025 [89]
		Homocysteine	Malik et al., 2017 [90]
	Lipid Metabolism	LDL Cholesterol, Apolipoprotein A1	Cao et al., 2021 [91]
Epigenetic	Non-Coding RNAs	microRNAs, Long Non-Coding RNAs	Wang et al., 2026 [92]

*LMNA—Lamin A/C (Prelamin A/C); FLNA—Filamin A; TPM4—Tropomyosin alpha-4 chain; ACTG1—Actin gamma 1; POSTN—Periostin; PAPPA—Pregnancy-Associated Plasma Protein-A; CFHR4—Complement Factor H-Related Protein 4; MBL2—Mannose-Binding Lectin 2*; LDL cholesterol—Low-density lipoprotein cholesterol.

**Table 2:** Circulating maternal biomarkers (miRNAs, lncRNAs) associated with CHD.

Study	Study Design	Cohort/Gestational Age at Sampling	CHD Diagnosis	Septal Defect	Maternal Serum Collection/Processing	Method/Assay	Biomarker Level	Result/AUC/Sensitivity/Specificity
Gu et al., 2019 [112]	Matched case–control	110 pregnant women (50 CHD, 60 controls); gestational age: 26–27 weeks	Fetal echocardiography and/or postnatal surgery and/or autopsy	VSD (*n* = 13)	5 mL of maternal venous blood; double centrifugation at 1600× *g* and 16,000× *g*, 4 °C; serum stored at −80 °C; RNA extracted using the mirVana PARIS kit; quality assessed by Nanodrop and gel electrophoresis	Microarray microRNA + validation RT-PCR	Down: *miR-142-5p*, *miR-4666a-3p*; Up: *miR-1275*, *miR-3664-3p*	microRNA: AUC 0.920, sensitivity 86%, specificity 92%; individually: *miR-142-5p* AUC 0.804, miR-1275 AUC 0.715, *miR-4666a-3p* AUC 0.703, *miR-3664-3p* AUC 0.694
Zhu et al., 2013 [110]	Multistage nested case–control study	60 pregnant women (30 CHD, 30 controls); gestational age: 22–28 weeks of gestation	Fetal echocardiography	VSD (*n* = 12), ASD (*n* = 4)	5 mL maternal venous blood; left 30–50 min at room temperature; centrifuged at 4000 rpm × 10 min; serum stored at −80 °C; RNA extracted using the mirVana PARIS Kit; cel-miR-39 spike-in used for normalization	SOLiD sequencing + qRT-PCR validation using TaqMan; 2-phase validation	Up: *miR-19b*, *miR-29c* (VSD); *miR-19b*, *miR-29c*, *miR-375* (ASD)	Panel of 4 microRNAs (*miR-19b*, *miR-22*, *miR-29c*, *miR-375*), AUC 0.813; individual sensitivity and specificity: *miR-19b* 74.1%/7.8%, *miR-29c* 63%/88.9%, *miR-22* 70.4%/66.7%, *miR-375* 55.6%/85.2%
Jin et al., 2021 [108]	Retrospective cohort	182 pregnant women (91 VSD, 91 controls); gestational age: 16–18 weeks of gestation	Fetal echocardiography	VSD (*n* = 91)	4 mL maternal venous blood; double centrifugation at 1600× *g* and 16,000× *g*; exosomes isolated using Ribo™ Exosome Isolation Reagent (Mölndal, Sweden); serum stored at −80 °C; RNA extracted from exosomes	Next-generation sequencing + qRT-PCR	Down: *hsa-miR-146a-5p*, *hsa-miR-199a-3* insignificant: *hsa-miR-181a-5p*, *hsa-miR-186-5p*	*hsa-miR-146a-5p*: AUC 0.997, sensitivity 98.1%, specificity 98.1%; *hsa-miR-199a-3p*: AUC 0.672, sensitivity 58.2%, specificity 99.9%
Xi et al., 2024 [109]	Case–control	72 pregnant women (31 CHD, 41 controls); gestational age: 22–34 weeks of gestation	Fetal echocardiography	ASD (*n* = 1) and VSD (*n* = 8)	3–4 mL maternal venous blood; centrifugation at 3000× *g* for 10 min; serum frozen at −80 °C; RNA extracted using TRIzol LS; quality assessed with NanoDrop	High-throughput sequencing + qRT-PCR validation	Down: *hsa-miR-3195, hsa-miR-122-5p*	*hsa-miR-3195*: AUC 0.675, *hsa-miR-122-5p*: AUC 0.702; combined AUC 0.729; sensitivity and specificity not reported separately
Wang et al., 2023 [104]	Observational, cohort retrospective	38 pregnant women (22 with fetal VSD, 16 controls); gestational age: 20–21 weeks of gestation	Prenatal fetal echocardiography + postnatal confirmation	VSD (*n* = 22)	5 mL maternal venous blood, collected in EDTA; centrifuged at 300× *g*	qPCR + RO	All downregulated lncRNAs: *LINC00598*, *LINC01551*, *GATA3-AS1*	Individual lncRNAs: *LINC00598* AUC 0.852, *LINC01551* AUC 0.864, *GATA3-AS1* AUC 0.957; combined 3-lncRNA panel AUC 0.994; sensitivity and specificity not reported individually
Gu et al., 2016 [111]	Paired case–control	124 pregnant women (62 CHD, 62 controls); gestational age: 24–28 weeks of gestation	Fetal echocardiography	VSD (*n* = 30), ASD (*n* = 18)	5 mL maternal venous blood (EDTA); centrifuged at 4000 rpm × 10 min; plasma stored at −80°C; RNA extracted using TRIzol LS; quality assessed with NanoDrop	Arraystar lncRNA microarray with qRT-PCR validation (normalized to GAPDH)	VSD: Upregulated: *ENST00000436681*; Downregulated: *AA584040, AA709223, BX478947* ASD: Downregulated: *AA709223*, *BX478947*	The five lncRNAs (*ENST00000436681*, *ENST00000422826*, *AA584040*, *AA709223*, *BX478947*) demonstrated significant diagnostic value
Turan et al., 2019 [113]	Prospective observational study	13 pregnant women (7 with mVSD, 6 controls; gestational age: 2nd trimester	Second-trimester fetal echocardiography	Isolated muscular VSD (*n* = 7)	Maternal serum from venous blood; RNA extracted from serum; expression analysis reported as molecules per 100 ng RNA	NanoString nCounter (profiling of 797 microRNAs)	Up (VSD): *hsa-miR-10a-5p*, *hsa-miR-1183*, *hsa-miR-1288-3p*, *hsa-miR-141-3p*, *hsa-miR-23c*, *hsa-miR-30d-5p*, hsa-*miR-375*, *hsa-miR-4451*, *hsa-miR-4455*, *hsa-miR-491-3p*, *hsa-miR-944*, *hsa-miR-95-3p*;	All microRNAs significantly upregulated compared to controls (*p* < 0.001)

CHD—congenital heart disease; VSD—ventricular septal defect; ASD—atrial septal defect; mVSD—muscular ventricular septal defect; RNA—ribonucleic acid; miRNA—microRNA; lncRNA—long non-coding RNA; RT-PCR—reverse transcription polymerase chain reaction; qRT-PCR—quantitative reverse transcription polymerase chain reaction; EDTA—ethylenediaminetetraacetic acid; RO—relative quantification/relative expression; NanoDrop—spectrophotometer for nucleic acid quantification; Trizol LS—reagent for RNA extraction from liquid samples; mirVana PARIS kit—commercial kit for RNA extraction; TaqMan—probe-based qPCR detection system; next-generation sequencing (NGS) —high-throughput RNA sequencing technology; high-throughput sequencing—sequencing method allowing simultaneous analysis of thousands of nucleic acid sequences; NanoString nCounter—platform for direct digital detection of nucleic acids; Arraystar—microarray platform for lncRNA profiling; AUC—area under the receiver operating characteristic (ROC) curve, indicator of diagnostic performance; RT—room temperature; SOLiD sequencing—sequencing by oligonucleotide ligation and detection, an NGS platform; GAPDH—glyceraldehyde 3-phosphate dehydrogenase, used as internal control/housekeeping gene in qRT-PCR; Spike-in cel-miR-39—exogenous control microRNA added for normalization in qRT-PCR experiments.

## Data Availability

No new data were created or analyzed in this study. Data sharing is not applicable to this article.

## Supplementary Materials

The following supporting information can be downloaded at https://www.mdpi.com/article/10.3390/biomedicines14030586/s1. File S1: PRISMA 2020 checklist.
