# Supplementary material for: Maternal Blood as a Window to the Fetal Heart: Novel Biomarkers for Early Detection of Septal Defects
Source: Biomedicines. 2026 Mar 5;14(3):586. doi: 10.3390/biomedicines14030586 (PMC13024671; doi:10.3390/biomedicines14030586)
Supplement: Supplementary file 1 [file biomedicines-14-00586-s001.zip › biomedicines-4158326-supplementary.pdf]

Preferred Reporting Items for Systematic reviews and Meta-Analyses extension for Scoping Reviews (PRISMA-ScR) Checklist

SECTION	ITEM	PRISMA-ScR CHECKLIST ITEM	REPORTED ON PAGE #
**TITLE**			
Title	1	Identify the report as a scoping review.	This report is presented as a scoping review investigating circulating maternal non-coding RNA biomarkers for the prenatal detection of congenital septal heart defects.
**ABSTRACT**			
Structured summary	2	Provide a structured summary that includes (as applicable): background, objectives, eligibility criteria, sources of evidence, charting methods, results, and conclusions that relate to the review questions and objectives.	This scoping review summarizes current evidence regarding maternal circulating microRNAs and long non-coding RNAs as potential non-invasive biomarkers for the prenatal detection of congenital heart defects, particularly septal defects. Electronic searches were conducted in PubMed, Scopus, and Web of Science for studies published between January 2010 and October 2025. Original research articles assessing RNA biomarkers in maternal blood were included. Seven studies met the eligibility criteria. Due to methodological heterogeneity, a narrative synthesis was performed. Several microRNAs and lncRNA panels demonstrated promising diagnostic performance, although variability in study design and small sample sizes limit clinical applicability. Further large-scale, standardized studies are required prior to clinical implementation.
**INTRODUCTION**			
Rationale	3	Describe the rationale for the review in the context of what is already known. Explain why the review questions/objectives lend themselves to a scoping review approach.	Congenital heart defects remain the most common congenital malformations worldwide. Although fetal echocardiography represents the mainstay of prenatal screening, its diagnostic performance may be limited in low-risk pregnancies and is operator-dependent. Emerging evidence suggests that maternal circulating non-coding RNAs may reflect molecular alterations involved in fetal cardiac

SECTION	ITEM	PRISMA-ScR CHECKLIST ITEM	REPORTED ON PAGE #
			development. Given the rapidly evolving and heterogeneous nature of this field, a scoping review approach was considered appropriate to map existing evidence, identify key biomarkers, and highlight research gaps.
Objectives	4	Provide an explicit statement of the questions and objectives being addressed with reference to their key elements (e.g., population or participants, concepts, and context) or other relevant key elements used to conceptualize the review questions and/or objectives.	The objective of this scoping review was to identify and summarize available evidence regarding maternal circulating microRNAs and long non-coding RNAs as potential non-invasive biomarkers for the prenatal detection of congenital septal defects (ventricular and atrial septal defects). The review focused on pregnant women (population), circulating RNA biomarkers (concept), and prenatal diagnosis of congenital heart disease (context).
**METHODS**			
Protocol and registration	5	Indicate whether a review protocol exists; state if and where it can be accessed (e.g., a Web address); and if available, provide registration information, including the registration number.	No prior protocol was registered for this review. The study was conducted in accordance with PRISMA reporting recommendations.
Eligibility criteria	6	Specify characteristics of the sources of evidence used as eligibility criteria (e.g., years considered, language, and publication status), and provide a rationale.	Eligible studies were original research articles published in English between January 2010 and October 2025. Studies assessing microRNA or long non-coding RNA expression in maternal blood samples in relation to fetal congenital heart defects were included. Reviews, case reports, case series, editorials, and studies evaluating non-maternal samples were excluded.
Information sources*	7	Describe all information sources in the search (e.g., databases with dates of coverage and contact with authors to identify additional sources), as well as the date the most recent search was executed.	A comprehensive literature search was conducted in PubMed, Scopus, and Web of Science. The final search was performed in November 2025.

SECTION	ITEM	PRISMA-ScR CHECKLIST ITEM	REPORTED ON PAGE #
Search	8	Present the full electronic search strategy for at least 1 database, including any limits used, such that it could be repeated.	The search strategy included combinations of the following keywords: “microRNA,” “lncRNA,” “congenital heart defects,” “ventricular septal defect,” “atrial septal defect,” “prenatal diagnosis,” and “non-invasive prenatal testing.” Search terms were adapted for each database.
Selection of sources of evidence†	9	State the process for selecting sources of evidence (i.e., screening and eligibility) included in the scoping review.	After removal of duplicates, titles and abstracts were screened for relevance. Full-text articles were assessed according to predefined inclusion and exclusion criteria. Seven studies were ultimately included. The selection process is illustrated in the PRISMA flow diagram
Data charting process‡	10	Describe the methods of charting data from the included sources of evidence (e.g., calibrated forms or forms that have been tested by the team before their use, and whether data charting was done independently or in duplicate) and any processes for obtaining and confirming data from investigators.	Data were extracted and charted using a structured approach. Extracted information included study design, sample size, type of congenital heart defect, type of RNA biomarker, laboratory methods, and reported diagnostic performance indicators. Due to heterogeneity, results were synthesized narratively.
Data items	11	List and define all variables for which data were sought and any assumptions and simplifications made.	The following variables were extracted: type of biomarker (microRNA or lncRNA), expression pattern (upregulated/downregulated), type of septal defect (VSD or ASD), gestational age at sampling, laboratory detection method (qRT-PCR, NGS, microarray, NanoString), and diagnostic performance (AUC, sensitivity, specificity).
Critical appraisal of individual sources of evidence§	12	If done, provide a rationale for conducting a critical appraisal of included sources of evidence; describe the methods used and how this information was used in any data synthesis (if appropriate).	A formal critical appraisal or risk-of-bias assessment was not performed, as the purpose of this scoping review was to map available evidence rather than to evaluate study quality.

SECTION	ITEM	PRISMA-ScR CHECKLIST ITEM	REPORTED ON PAGE #
Synthesis of results	13	Describe the methods of handling and summarizing the data that were charted.	Given the methodological heterogeneity across studies, including differences in cohort characteristics, RNA extraction methods, and outcome reporting, a quantitative meta-analysis was not feasible. Therefore, a structured narrative synthesis was conducted.
**RESULTS**			
Selection of sources of evidence	14	Give numbers of sources of evidence screened, assessed for eligibility, and included in the review, with reasons for exclusions at each stage, ideally using a flow diagram.	Seven studies met the inclusion criteria and were included in the final synthesis. The study selection process is presented in the PRISMA flow diagram.
Characteristics of sources of evidence	15	For each source of evidence, present characteristics for which data were charted and provide the citations.	Included studies consisted of case-control, retrospective cohort, and prospective observational designs. Sample sizes ranged from 13 to 182 participants. Most studies focused on ventricular septal defects, while some included atrial septal defects. Maternal serum, plasma, or exosomal RNA was analyzed using various molecular techniques.
Critical appraisal within sources of evidence	16	If done, present data on critical appraisal of included sources of evidence (see item 12).	Not applicable, as no formal critical appraisal was conducted.
Results of individual sources of evidence	17	For each included source of evidence, present the relevant data that were charted that relate to the review questions and objectives.	Several individual microRNAs and lncRNAs demonstrated promising diagnostic performance. For example, hsa-miR-146a-5p showed high diagnostic accuracy for VSD detection, while panels of lncRNAs such as LINC00598, LINC01551, and GATA3-AS1 achieved high AUC values. Other studies identified panels of microRNAs associated with septal defects.
Synthesis of results	18	Summarize and/or present the charting results as they relate to the review questions and objectives.	Overall, the evidence suggests that maternal circulating microRNAs and lncRNAs may serve as potential biomarkers for the prenatal detection of septal defects. However, variability in biomarker selection, analytical

SECTION	ITEM	PRISMA-ScR CHECKLIST ITEM	REPORTED ON PAGE #
			methods, and population characteristics limits comparability across studies.
**DISCUSSION**			
Summary of evidence	19	Summarize the main results (including an overview of concepts, themes, and types of evidence available), link to the review questions and objectives, and consider the relevance to key groups.	This review highlights emerging evidence supporting the potential role of maternal circulating RNA biomarkers in prenatal CHD detection. Several candidate biomarkers demonstrated promising diagnostic performance. However, most studies were conducted in limited populations and exhibited methodological heterogeneity.
Limitations	20	Discuss the limitations of the scoping review process.	Limitations include small sample sizes, heterogeneity in laboratory techniques and study design, absence of standardized protocols, and lack of formal quality appraisal. Additionally, most studies were conducted in specific geographic regions, limiting generalizability.
Conclusions	21	Provide a general interpretation of the results with respect to the review questions and objectives, as well as potential implications and/or next steps.	Maternal circulating microRNAs and long non-coding RNAs represent promising non-invasive biomarkers for the early detection of congenital septal defects. Nevertheless, further large-scale, multicenter studies using standardized methodologies are required before clinical implementation can be considered.
**FUNDING**			
Funding	22	Describe sources of funding for the included sources of evidence, as well as sources of funding for the scoping review. Describe the role of the funders of the scoping review.	This research received no external funding. The authors declared no conflicts of interest. No specific funding sources were reported for the included studies within the scope of this review.
registration number**https://osf.io/qgb89**			

JBI = Joanna Briggs Institute; PRISMA-ScR = Preferred Reporting Items for Systematic reviews and Meta-Analyses extension for Scoping Reviews.
* Where *sources of evidence* (see second footnote) are compiled from, such as bibliographic databases, social media platforms, and Web sites.
† A more inclusive/heterogeneous term used to account for the different types of evidence or data sources (e.g., quantitative and/or qualitative research, expert opinion, and policy documents) that may be eligible in a scoping review as opposed to only studies. This is not to be confused with *information sources* (see first footnote).

‡ The frameworks by Arksey and O’Malley (6) and Levac and colleagues (7) and the JBI guidance (4, 5) refer to the process of data extraction in a scoping review as data charting.

§ The process of systematically examining research evidence to assess its validity, results, and relevance before using it to inform a decision. This term is used for items 12 and 19 instead of "risk of bias" (which is more applicable to systematic reviews of interventions) to include and acknowledge the various sources of evidence that may be used in a scoping review (e.g., quantitative and/or qualitative research, expert opinion, and policy document).

From: Tricco AC, Lillie E, Zarin W, O'Brien KK, Colquhoun H, Levac D, et al. PRISMA Extension for Scoping Reviews (PRISMAScR): Checklist and Explanation. Ann Intern Med. 2018;169:467–473. doi: 10.7326/M18-0850.
